# Supplementary material for: Changes in neural progenitor lineage composition during astrocytic differentiation of human iPSCs
Source: eLife. 2025 Oct 28;13:RP96423. doi: 10.7554/eLife.96423 (PMC12563561; doi:10.7554/eLife.96423)
Supplement: Supplementary file 2. [file elife-96423-supp2.docx]

**Supplementary file 2. Antibodies used in this study.**

| Target | Species | Producer | Catalogue number | Dilution for use |
| --- | --- | --- | --- | --- |
| ALDH1L1 | MS | Abcam | ab56777 | 1:500 |
| AQP4 | RB | Abcam | ab259318 | 1:200 |
| BFP | rabbit | Evrogen | AB233 | 1:3000 |
| CD44 | mouse | Cell Signalling Technology | 3750 | 1:400 |
| EAAT2 | goat | Abcam | ab235202 | 1:200 |
| FOXA2 | goat | R&D system | AF2400 | 1:1000 |
| GFAP | rat | Life Tech | AB10533 | 1:1000 |
| LMX1A | rabbit | Millipore | HPA030088 | 1:2000 |
| NESTIN | mouse | BD | BD611659 | 1:300 |
| NFIA | rabbit | Abcam | AB228897 | 1:1000 |
| NKX6.1 | mouse | DSHB | F55A10 | 1:100 |
| OTX2 | goat | R&D system | AF1979 | 1:200 |
| PAX6 | mouse | DSHB | PAX6 | 1:1000 |
| S100B | rabbit | Abcam | ab52642 | 1:1000 |
| SOX9 | goat | R&D system | AF3075 | 1:300 |
| Anti-Goat IgG, Alexa Fluor 488 | donkey | Invitrogen | A32814 | 1:1000 |
| Anti-Goat IgG, Alexa Fluor 555 | donkey | Invitrogen | A32816 | 1:1000 |
| Anti-Goat IgG, Alexa Fluor 647 | donkey | Invitrogen | A32849 | 1:1000 |
| Anti-Mouse IgG, Alexa Fluor 488 | donkey | Invitrogen | A21202 | 1:1000 |
| Anti-Mouse IgG, Alexa Fluor 555 | donkey | Invitrogen | A31570 | 1:1000 |
| Anti-Mouse IgG, Alexa Fluor 647 | donkey | Invitrogen | A31571 | 1:1000 |
| Anti-Rabbit IgG, Alexa Fluor 488 | donkey | Invitrogen | A21206 | 1:1000 |
| Anti-Rabbit IgG, Alexa Fluor 555 | donkey | Invitrogen | A31572 | 1:1000 |
| Anti-Rabbit IgG, Alexa Fluor 647 | donkey | Invitrogen | A31573 | 1:1000 |
| Anti-Rat IgG, Alexa Fluor 488 | donkey | Invitrogen | A21208 | 1:1000 |

**Supplementary Table 2. Settings used for processing published datasets.**

| PMID | Tissue | Age | Scale factor | PCs used | Harmony integration | Clustering resolution | Re-annotation |
| --- | --- | --- | --- | --- | --- | --- | --- |
| 32826893 | SN and cortex | Adult | 10000 | 50 | No | 0.5 | No |
| 33723434 | multiple | CS22 | 10000 | 50 | Yes | 0.3 | Yes |
| 34616070 | multiple | GW25 | 10000 | 50 | Yes | 0.4 | Yes |
| 32923614 | Neocortex | GW9-28 | 100000 | 30 | Yes | 0.9 | Yes |
| 31303374 | Neocortex | GW17-18 | 10000 | 30 | Yes | 0.5 | Yes |
| 29539641 | Prefrontal cortex | GW8-26 | 100000 | 30 | Yes | 0.9 | Yes |
| 28817799 | PSC-derived astrocytes | - | 10000 | 30 | No | 0.3 | No |
| 27716510 | Ventral midbrain | GW7-11 | 10000 | 30 | No | 0.5 | No |

# Supplementary Methods

## Immunocytochemistry

For nuclear antigen detection, an additional fixation with methanol gradient was performed, which include 5 mins each in 33% and 66% methanol at room temperature followed by 100% methanol for 20 min at -20°C. Cultures were then returned to PBST via inverse gradient and were then permeabilized with three 10-minute washes in 0.3% Triton-X-100 PBS (PBS-T) and then blocked in PBS-T containing 1% BSA and 3% donkey serum. Cells were incubated with primary antibodies in blocking solution overnight at 4°C. Following three PBS-T washes, Alexa-Fluor secondary antibodies (Thermo Fisher Scientific) were added at 1:1000 PBS-T for 1 hour at ambient temperature in the dark. Three PBS-T washes were then performed that included once with DAPI (Molecular Probes).

## Single-cell RNA-sequencing analysis

In batch 1 (chip B), CD49f^+^ astrocytes were purified using FACS. For BFP^+^ astrocytes, BFP expression was also a sorting criterion. In batch 2 (chip E), unsorted astrocytes, a sample of FAC-sorted BFP^+^/CD49f^+^ astrocytes, and a sample of iPSC-derived neurons were included. Minimum transcriptomic difference was observed between FAC-sorted BFP^+^/CD49f^+^ and unsorted BFP^+^ astrocytes, with only 154 genes significantly differentially expressed between the two.

The libraries of batch 1 (chip B) were split into two lanes of the same flow cell following the NovaSeq Xp Workflow. FASTQ files of samples on chip B from the two lanes were merged by appending the FASTQ file from one lane to the read-matched and sample-matched FASTQ file of another lane. The quality of the FASTQ files was assessed using FastQC 0.11.8 and summarized using multiqc 1.7. Using the Cogent NGS Analysis Pipeline software v 1.5.1 (Takara), FASTQ files containing all indices for each chip were demultiplexed to FASTQ files containing one index per file. Adaptor sequences were removed using cutadapt 3.2 with the following settings: -m 15 --trim-n --max-n 0.7 -q 20. Trimmed FASTQ files were aligned to the *Homo sapiens* GRCh38.106 primary assembly with the BFP reporter gene attached to the end of the genome, using STAR 2.7.9a (Dobin et al. 2013) with the following settings: --outSAMtype BAM Unsorted --quantMode TranscriptomeSAM --outReadsUnmapped Fastx --outSAMstrandField intronMotif --chimSegmentMin 12 --chimJunctionOverhangMin 8 --chimOutJunctionFormat 1 --alignSJDBoverhangMin 10 --alignMatesGapMax 100000 --alignIntronMax 100000 --alignSJstitchMismatchNmax 5 -1 5 5 --chimMultimapScoreRange 3 --chimScoreJunctionNonGTAG -4 --chimMultimapNmax 20 --chimNonchimScoreDropMin 10 --peOverlapNbasesMin 12 --peOverlapMMp 0.1 –alignInsertionFlush Right --alignSplicedMateMapLminOverLmate 0 --alignSplicedMateMapLmin 30. The genome index was also built using the same version of STAR. After alignment, gene-level quantification was performed using featureCounts from subread 2.0.0 (Liao et al. 2013) with the following settings: -t exon --primary -R CORE -F GTF -Q 0 -B -g gene_id. The count matrix of each index was combined in R 4.2.0.

Gene-level filtering was applied by including only protein-coding genes with at least five total counts across all cells and being expressed in at least 1% of all cells. Poor quality cells were then identified using the is.outlier function from the scater 1.28.0 (McCarthy et al. 2017). Poor quality cells were defined as having a high percentage of mitochondrial gene count, or high or low the total number of genes detected, or high total gene counts. The thresholds of each metrics for each sample were determined as twice the median absolute deviation of the sample. Raw gene counts were log normalised with a scale.factor setting of 1×10^5^. Data from the two batches of experiments were integrated using the *FindIntegrationAnchors* and *IntegrateData* based on the top 2000 common highly variable genes and the first 30 dimensions of principal components. The percentage of mitochondrial gene count and total gene count were regressed out using the *ScaleData* function. Principal component analysis (PCA) was performed on the top 2000 high variable genes and the number of principal components used for uniform manifold approximation and projection (UMAP) was determined using the JackStraw method. UMAP and unbiased Louvain clustering was performed on the first 33 principal components. One versus all DEG analysis was performed using the *FindAllMarkers* function in Seurat using the MAST method with Chip as the latent variable. Pairwise differential gene expression analysis was performed using the *FindMarkers* function in Seurat using the MAST method with Chip as the latent variable. Gene ontology (GO) enrichment analysis was performed using a hypergeometric test with a background of 17478, and the GO term database was downloaded using the *org.Hs.eg.db* package. Revigo v1.8.1 was used to group the representative GO terms based on semantic similarity using a size setting of 0.5, Homo sapiens database, and SimRel method for semantic similarity (Schlicker et al. 2006; Supek et al. 2011).

The raw gene counts of all published datasets were downloaded from Gene Expression Omnibus and processed in R4.2.0. Gene level filtering was performed by retaining only protein-coding genes with more than five total counts across all cells. Gene counts were normalised using the NormalizeData function (scale.factor settings are listed in Supplementary Table 2). PCA was performed based on the top 2000 highly variable genes to obtain the first 50 PCs. Visual inspection of the elbow plot was used to determine the number of PCs for downstream analysis. Batch effect between subjects was evaluated on the two-dimensional PC2~PC1 plot. Where inter-subject batch effect was observed, Harmony integration was performed based on the PCs selected in the previous step (Supplementary Table 2). UMAP was performed based on either the PCA or Harmony reduction (using the top 30 dimensions), and Louvain clustering was performed (settings shown in Supplementary Table 2). Cluster identities were verified against the reported annotation and re-annotated where necessary (no annotation or astrocyte identity reported) based on the the expression of known markers.

We observed that cells mostly segregated on UMAP plots based on chips, indicating a potential batch effect (Figure S5D and S5F). After integration, samples from different chips mostly mix with each other (Figure S5E), and CD49f^+^ sorted BFP^+^ astrocyte samples on two chips derived from the same batch of astrocyte differentiation clustered together on UMAP plot, indicating successful integration (Figure S5F-G).

Dobin, A. et al. 2013. STAR: ultrafast universal RNA-seq aligner. *Bioinformatics* 29(1), pp. 15-21. doi: 10.1093/bioinformatics/bts635

Liao, Y., Smyth, G. K. and Shi, W. 2013. The Subread aligner: fast, accurate and scalable read mapping by seed-and-vote. *Nucleic Acids Res* 41(10), p. e108. doi: 10.1093/nar/gkt214

McCarthy, D. J., Campbell, K. R., Lun, A. T. L. and Wills, Q. F. 2017. Scater: pre-processing, quality control, normalization and visualization of single-cell RNA-seq data in R. *Bioinformatics* 33(8), pp. 1179-1186. doi: 10.1093/bioinformatics/btw777

Schlicker, A., Domingues, F. S., Rahnenführer, J. and Lengauer, T. 2006. A new measure for functional similarity of gene products based on Gene Ontology. *BMC Bioinformatics* 7(1), p. 302. doi: 10.1186/1471-2105-7-302

Supek, F., Bošnjak, M., Škunca, N. and Šmuc, T. 2011. REVIGO Summarizes and Visualizes Long Lists of Gene Ontology Terms. *PLOS ONE* 6(7), p. e21800. doi: 10.1371/journal.pone.0021800
