# Supplementary material for: Changes in neural progenitor lineage composition during astrocytic differentiation of human iPSCs
Source: eLife. 2025 Oct 28;13:RP96423. doi: 10.7554/eLife.96423 (PMC12563561; doi:10.7554/eLife.96423)
Supplement: Supplementary file 3. [file elife-96423-supp3.docx]

**Supplementary file 3. Settings used for processing published datasets**

| **PMID** | **Tissue** | **Age** | **Scale factor** | **PCs used** | **Harmony integration** | **Clustering resolution** | **Re-annotation** |
| --- | --- | --- | --- | --- | --- | --- | --- |
| 32826893 | SN and cortex | Adult | 10000 | 50 | No | 0.5 | No |
| 33723434 | multiple | CS22 | 10000 | 50 | Yes | 0.3 | Yes |
| 34616070 | multiple | GW25 | 10000 | 50 | Yes | 0.4 | Yes |
| 32923614 | Neocortex | GW9-28 | 100000 | 30 | Yes | 0.9 | Yes |
| 31303374 | Neocortex | GW17-18 | 10000 | 30 | Yes | 0.5 | Yes |
| 29539641 | Prefrontal cortex | GW8-26 | 100000 | 30 | Yes | 0.9 | Yes |
| 28817799 | PSC-derived astrocytes | - | 10000 | 30 | No | 0.3 | No |
| 27716510 | Ventral midbrain | GW7-11 | 10000 | 30 | No | 0.5 | No |
